# Inhibition of Topoisomerases by Metal Thiosemicarbazone Complexes

**DOI:** 10.3390/ijms241512010

**Published:** 2023-07-27

**Authors:** Xiaohua Jiang, Lauren A. Fielding, Hunter Davis, William Carroll, Edward C. Lisic, Joseph E. Deweese

**Affiliations:** 1 Department of Chemistry, Vanderbilt University, Nashville, TN 37240, USA; 2 Department of Biological, Physical and Human Sciences, Freed Hardeman University, Henderson, TN 38340, USA; 3 Department of Chemistry, Tennessee Tech University, Cookeville, TN 38505, USA; 4 Department of Biochemistry, Vanderbilt University School of Medicine, Nashville, TN 37240, USA

**Keywords:** thiosemicarbazone, topoisomerase, antitumor, bis-thiosemicarbazone

## Abstract

Topoisomerases, common targets for anti-cancer therapeutics, are crucial enzymes for DNA replication, transcription, and many other aspects of DNA metabolism. The potential anti-cancer effects of thiosemicarbazones (TSC) and metal–TSC complexes have been demonstrated to target several biological processes, including DNA metabolism. Human topoisomerases were discovered among the molecular targets for TSCs, and metal-chelated TSCs specifically displayed significant inhibition of topoisomerase II. The processes by which metal–TSCs or TSCs inhibit topoisomerases are still being studied. In this brief review, we summarize the TSCs and metal–TSCs that inhibit various types of human topoisomerases, and we note some of the key unanswered questions regarding this interesting class of diverse compounds.

## 1. Introduction

Topoisomerases (Tops) are essential enzymes for genome stability that are involved in DNA metabolism through the maintenance of DNA topology. All cells maintain DNA supercoiling in a dynamic process that is required for transcription, replication, and cell division to take place. Tops are found across all domains of life and several viruses encode topoisomerases. Tops are divided into two families: Type I and Type II.

Type I Tops cut and religate one strand of the double helix coupled with either a strand passage or controlled rotation mechanism to remove DNA positive (overwinding) and negative (underwinding) supercoiling. To modulate supercoiling, remove DNA knots, and unlink catenated DNA (intertwined DNA), Type II Tops form a double-stranded DNA break and passes a double-strand DNA section through the break before ligating the DNA back together. In both mechanisms, the DNA break is stabilized through a covalent intermediate with an active site tyrosine residue on the Top. There are six human Tops including Top1B, mitochondrial Top1B, Top2α, Top2β, Top3α, Top3β [1].

There are two main Type I subfamilies IA (Top3α/β in humans) and IB (Top1B/mitochondrial Top1B in humans) [1,2]. The subfamilies differ both structurally and mechanistically, and these differences have been reviewed elsewhere [1,2]. There is a Type IC that is only found in an archaeal species.

There are two main Type II subfamilies, which include Type IIA and Type IIB depending on either a 4- or 2-base stagger between the cleaved positions on the DNA, respectively [1,3,4,5]. Human Top2α and Top2β both belong to the Type IIA subfamily [1,5]. While the names and structures for the archaeal and bacterial counterparts differ, this review will focus on the mammalian forms of the eukaryotic enzymes.

During the catalytic cycle, Tops form temporary single- or double-stranded DNA breaks [1,5]. These momentary DNA breaks may become permanent, leading to DNA damage and cell death [1,2,4]. Several widely-used anti-cancer therapeutics target either Type I or Type II Tops, and in a similar manner, several antibacterial agents are used to target bacterial Tops to fight off infections. Interfacial poisons and catalytic inhibitors are the two primary groups of inhibitors for Top enzymes [2,4,6]. Whereas catalytic inhibitors prevent Tops from completing its catalytic cycle, interfacial poisons stabilize single-stranded or double-stranded breaks leading to further damage [2,4,6]. The general mechanism for catalytic inhibitors is interaction with the N-terminal ATPase domain, which is a clamp-like region at the “top” of the enzyme. Top poisons are thought to act by a “doorstop” mechanism where the drug molecule prevents ligation by slipping between the cleaved ends of DNA during catalysis. In general, poisons tend to lead to accumulation of cleaved DNA while catalytic inhibitors do not. In addition, some agents are reactive and lead to inactivation of the enzyme and/or covalent adduction, which could occur either in the active site or at or near to the ATPase domain [6,7].

Thiosemicarbazones (TSCs) are a varied class of compounds with common N-N-S coordinates. They were discovered in the 1950s with anti-bacterial, anti-fungal, and anti-tumor activities [8,9]. TSCs are multi-target drugs and the molecular mechanisms involved metal chelation, DNA interference, topoisomerase inhibition and ribonucleotide reductase inhibition [10]. Many metal–TSC complexes have been synthesized and were found to be more effective in cell toxicity experiments than the ligand form of the TSC [10,11]. The cell toxicity of metal–TSCs included a decrease in de novo purine synthesis and inhibition of IMP dehydrogenase, DNA polymerase activity, and topoisomerase II activity [10,11,12]. Recent studies in yeast have uncovered other cellular mechanisms for metal-bis(TSC), which includes chromatin remodeling, cytoskeleton organization, mitochondrial function, and iron metabolism [13]. Due to the varied structures of TSCs, they impact multiple cellular targets. In this paper, we summarize the TSCs that are targeting Tops with a particular emphasis on metal–TSCs.

**Figure 1:**
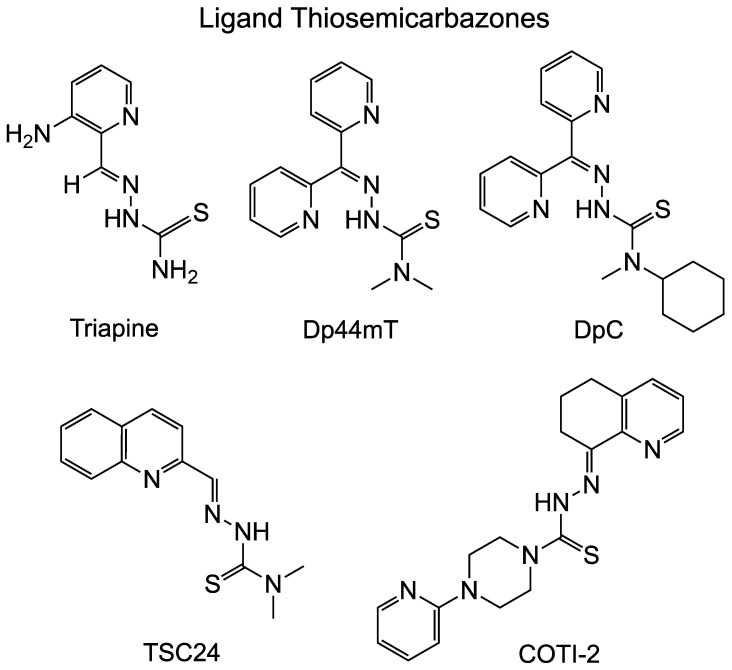
Ligand TSC compounds. Examples of several ligand TSCs are shown including some that have undergone clinical or pre-clinical trials. Some of these are known to chelate metals in cellular contexts. Structures prepared using ChemDraw 20.1.

Several examples of ligand TSCs that have been studied either clinically or in animal models are shown in Figure 1. Triapine (3-aminopyridine-2-carboxaldehyde thiosemicarbazone; 3-AP) is the first thiosemicarbazone to be approved for clinical trials. It inhibits ribonucleotide reductase and chelates iron to kill cancer cells. There are more than 30 Phase I and Phase II clinical trials with Triapine, but it has disadvantages of adverse events, such as methemoglobinemia, and a short plasma half-life [14]. In recent years, TSCs gained more attention as potential anticancer drugs since they impact multiple potential targets. Polypharmacology is particularly useful in the metastasis stage of cancer treatment. Dp44mT, (di-2-pyridylketone-4,4-dimethyl-3-thiosemicarbazone) has potent and broad antitumor activities in a panel of human xenografts in nude mice [15]. It not only chelates iron but also has redox properties, similar to Triapine [15]. Dp44mT showed conflicting results in Top inhibition, which is discussed later in this article. DpC, (di-2-pyridylketone 4-cyclohexyl-4-methyl-3-thiosemi-carbazone), optimized based on the structure of Dp44mT, has superior activity against human pancreatic cancer xenografts in nude mice [16].

COTI-2 [4-(2-pyridinyl)-2-(6,7-dihydro-8(5H)-quinolinylidene)-hydrazide], a TSC ligand, was identified through in silico screening and was found to inhibit many human cell lines in vivo [17]. COTI-2 has been shown to have anticancer activities through p53-dependent and p53-independent mechanisms [18]. Bis(thiosemicarbazone) metal complexes have been recently applied to neurodegenerative disease since they are able to restore metal balance in neurons. They showed promising results in animal models for Alzheimer’s disease, Parkinson disease, and amyotrophic lateral sclerosis [19].

## 2. Thiosemicarbazones as Inhibitors of Topoisomerases

### 2.1. TSC Ligand or bis(TSC) Ligand Inhibition of Topoisomerases

TSCs have been studied for decades for antiviral, antifungal and antiproliferation activity (Table 1). In recent years, Triapine (Figure 1), a member of TSC family, has been studied in phase I and II clinical trials [20,21,22,23]. The major molecular target of Triapine was identified as ribonucleotide reductase [23,24]. Triapine showed some inhibition of Top2A but only in the presence of Cu(II) [25]. Previous work on Triapine did not show any inhibition of relaxation or poisoning of plasmid DNA cleavage with Top2α [26].

In a series of TSC compounds, Top2 was discovered as one of the molecular targets for the ligand TSC24 (Figure 1) [27]. TSC24 showed high potent activity with an IC_50_ of 0.02 μM against the HT-29 cell line [27]. TSC24 inhibited tumor growth of S-180 sarcoma-bearing mice in a dose-dependent manner, with inhibitory rates of 17.6%, 35.8%, and 76.7% at doses of 12.5, 25, and 50 (mg/kg)/day, respectively, after it was administered intraperitoneal (IP) for 7 days [21].

The pattern of TSC antiproliferative effects against the human cancer cell line (NCI-60) is similar to those found with recognized anticancer treatments [27]. These investigations revealed TSC24’s profile is comparable to known Top2 agents. TSC24 was further investigated and it was found that it hindered DNA relaxing and decatenation by inhibiting the Top2α ATPase domain [27].

Another TSC ligand that has been well studied is Dp44mT (Figure 1). It induces G1 cell cycle arrest and reduces cancer cell clonogenic growth in the breast cancer line MDA-MB-231 at nanomolar concentrations [22]. In relaxation and cleavage experiments, Dp44mT was shown to preferentially target Top2α, with minimal effect on Top2β and little inhibition of Top1 [28]. But this result is controversial as an additional study found that the ligand Dp44mT did not inhibit Top2α or increase cellular cleavage complexes [26]. A more recent study on the ligand Dp44mT also found little effect of the compound alone, but they found inhibition when combined with Cu(II) [25]. The cell toxicity result is consistent with previous studies [22] and IC_50_ against the cancer cell line is in a nanomolar concentration. The Cu(II) complex of Dp44mT showed similar or lower IC_50_ compared with Dp44mT [25]. It is unclear whether it was the presence of Cu(II) alone or if the Cu(II) formed a complex with the ligand leading to the effect in this case. As is discussed below, it is possible that the ligand forms alone show little activity, while metal-chelated forms have varying levels of activity depending on the metal ion.

Computational docking and surface plasmon resonance studies support the ability of TSC24 to bind near the ATP binding pocket, but it is unclear if this is generalizable to other TSCs and whether this has been biochemically validated. Both TSC24 and Dp44mT appear to act as catalytic inhibitors, and there is an increase in DNA cleavage seen with Dp44mT [27,28]. TSC24 does not appear to increase strand breaks in cells, nor block the effects of VP16 [27].

A series of thiosemicarbazones and 4-thiazolindinones have been synthesized and some were identified with activity against Top2. The thiosemicarbazone (E)-2-(1H-indol-3ylmethylene)-*N*-(naphthalen-1-yl) hydrazinecarbothioamide (compound **2b**) has a IC_50_ of 0.01 µM against colorectal adenocarcinoma (HT-29) and leukemia (K562) cells and appear to weakly inhibit plasmid DNA relaxation by Top2α [29]. Compound **3a** [(*Z*)-2-(acridin-9-ylmethylene)-*N*-phenyl-hydrazinecarbothioamide)] and (compound **3h**) [(*Z*)-2-(acridin-9-ylmethylene)-*N*-(naphtalen-1-yl) hydrazinecarbothioamide] also had a limited ability to inhibit relaxation [30]. Detailed studies on the structure–activity relationship of TSCs against Top2α showed that most TSC ligands inhibit Top2α very weakly or not at all (Table 1) [31,32,33,34].

Despite the fact that TSC ligands were initially found to target Top2, the majority of the ligands produced had little or no effect on Top1 [35] or Top2 [32,33,34,36,37]. Metal–TSCs, on the other hand, showed profound inhibition on Tops, as discussed below.

**Table 1:** TSC ligand inhibition on Tops.

Name	Inhibition of Top	Reference
TSC24	Inhibits Top2α DNA relaxation at 25 µM Inhibits Top2 at decatenation at 100 µM	[27]
Dp44mT	Inhibits human Top2α in relaxation assay with 5′ labeled 161-bp fragment from pBluescript SK phagemid DNA Does not inhibit human Top2β or human TopoI	[28]
Dp44mT	Does not inhibit Top2α in decatenation Does not increase cleavage complex by human Top2α	[26]
Triapine		
Compound **2b**	Inhibits human Top2α weakly at 100 µM	[29]
Compound **3a**	Inhibit human Top2α-mediated DNA relaxation at 100 µM	[30]
Compound **3h**		
Triapine	Do not inhibit Top2α-mediated DNA relaxation at 50 µM	[25]
Dp44mT		
Compound **24**	Do not inhibit isolated Top2 from L1210	[12]
Compound **36**		
NQTS	Does not inhibit Top2α	[36]
HFp4mT, HFp4pyrrT	Inhibit Top2α at 100 µM	[37]
HFp4eT, HFp4ipT, HFp4alT, HAp4mT, HAp4-eT, HFp4bzT, HFpT, HAp4alT, and HApz4mT	Do not inhibit Top2α at 100 µM	[37]
BZP–TSC ligands series	Inhibits Top2α slightly at 50 µM	[20]
ATZ ligand series	Do not inhibit Top2α at 10 µM, do not increase cleavage complex	[33]
BZP ligands series		
APY	Does not inhibit Top2α at 100 µM	[32]
APY	Does not inhibit Top2β at 200 µM	[34]
APZ	Inhibits Top2α at 100 µM	[32]
BZP	Inhibits Top2β at 200 µM weakly	[34]
HPyCT4BrPh	Does not inhibit human Top1B at 50 µM	[35]

### 2.2. Metal–TSC or Metal–Bis(TSC) Inhibition of Topoisomerases

TSCs can chelate with various metal ions. Metal–TSC complexes have been produced from copper (Cu), nickel (Ni), palladium (Pd), ruthenium (Ru), tin (Sn), gallium (Ga), gold (Au), and cobalt (Co) [31,36,38,39,40,41,42]. Metal–TSCs inhibit Tops more strongly than their TSC ligands [25,31,32,33,34,37]. As will be discussed more below, metal–TSCs appear to be active against both Type I (Table 2) and Type II Tops (Table 3).

#### 2.2.1. Inhibition of Type I Top

**Figure 2:**
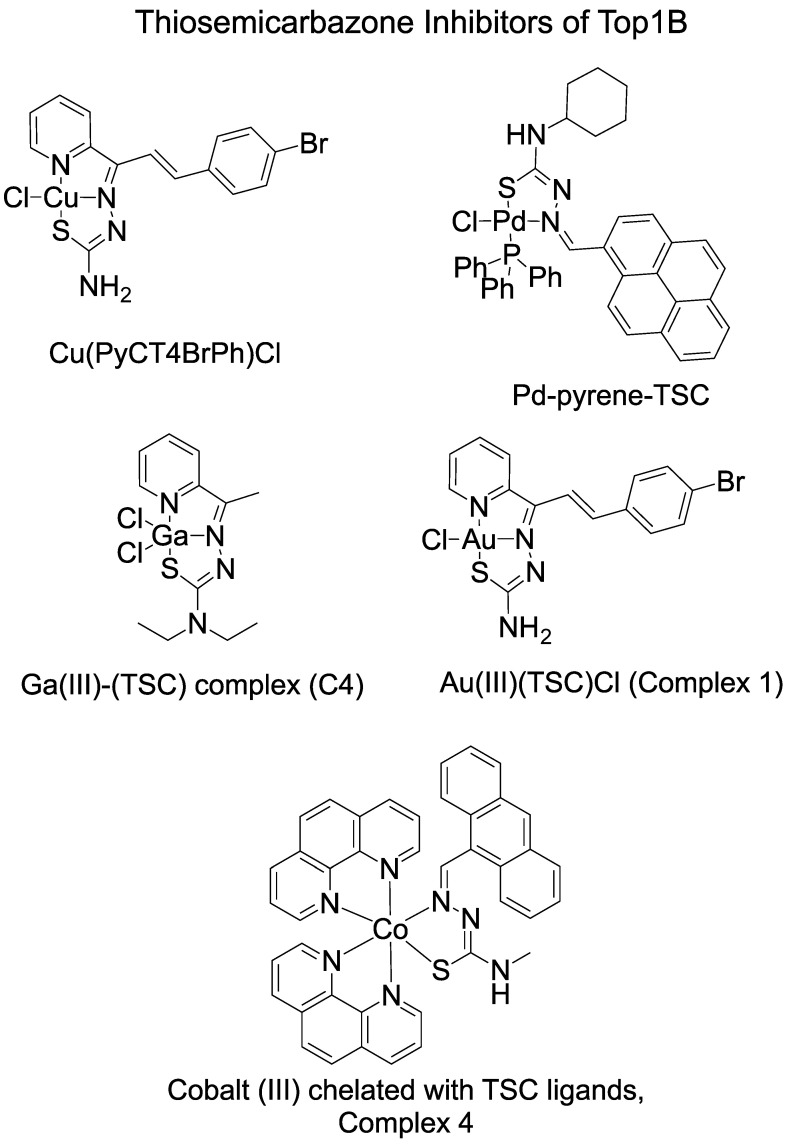
Examples of metal–TSC complexes studied with Top1B. These compounds were found to display varying activity against Top1B and include various metal ions.

Human Top1B belongs to the Type I Top family. It relaxes the DNA supercoils during DNA replication, recombination, and transcription by cutting one strand of DNA and performing a controlled rotation/swivel mechanism [1,2]. Top1B inhibitors are a class of compounds that target the enzyme and prevent it from relaxing DNA, leading to the accumulation of DNA damage. These inhibitors have potential as antitumor agents since tumor cells are under fast proliferation and DNA replication, making them more susceptible to DNA damage. Several classes of Top1B inhibitors have been developed, including camptothecin analogs and indolocarbazoles [1,2,46]. There are several metal–TSC compounds that have been studied against Top1B activity (Table 2 and Figure 2).

Cu(PyCT4BrPh)Cl [Cu(3-(4-bromophenyl)-1-pyridin-2-ylprop-2-en-1-one-thiosemi-carbazone)Cl] was studied against human Top1B [35]. It inhibited Topo1B by partially blocking ligation of the cleaved DNA [35]. The complex also reduced enzyme-DNA binding according to an EMSA assay [35]. Additional studies are needed to clarify the exact mechanism. The cytotoxicity of Cu(HPyCT4BrPh) increased 6-fold against THP-1 (IC_50_ = 0.20 µM) and 8-fold against MCF-7 cells (IC_50_ = 0.16 µM) compared with ligand HpyCT4BrPh [29].

In another study, pyrene TSCs were complexed with Pd (Complex 1) and examined for inhibition of human Top1B [43]. It has an IC_50_ of 7.59 µM in A2780 human ovarian carcinoma cells [38]. Its IC_50_ for A2780 cisplatin resistant human ovarian carcinoma cells is even lower with a value of 3.16 µM [43]. Pd–pyrene–TSC complexes inhibited relaxation of supercoiled plasmid by human Top1B at 12.5 µM [43]. Additionally, the Pd–pyrene–TSC complex displayed the ability to inhibit ligation of cleaved DNA with Top1B, similar to Cu(PyCT4BrPh)Cl [43].

One group reported the use of a Ga(III)–TSC complex, [*N*,*N*-diethyl-2-[1-(2-pyridinyl) ethylidene]hydrazinecarbothioamide-*N*,*N*,S-gallium(III)]bis(chloride), referred to as C4 in the study [42]. Based upon their results, human Top1B cleavage activity was inhibited by the Ga(III)–TSC complex while the ligand alone did not show significant inhibition [42]. C4 showed selective activities against tumor cells. It exhibited an IC_50_ of 0.30µM for lung cancer cell line NCI-H460 cells, 0.35 µM for T24 cells of the urinary bladder cancer cell line, 0.55 µM for BEL-7402 human liver cancer, and 0.76 µM for MSTO-211H, human mesothelioma cell line, while it showed low cell toxicity to the normal cell line—human fetal lung fibroblast cells with an IC_50_ higher than 28.65 µM [36].

The Au(III)–TSC complex [(3-(4-bromophenyl)-1-pyridin-2-ylprop-2-en-1-enone thiosemicarbazonato)chlorogold(III)] chloride, [Au(PyCT4BrPh)Cl]Cl, was studied with human Top1B and found to inhibit relaxation at 1.5 μM [31]. In contrast, HAuCl_4_·3H_2_O did not inhibit until 200 µM. Pre-incubation of Top1B with this compound increased the inhibition, which suggests gold(III)–TSC binds and inhibits the activity of Topo1B [31]. The gold(III)–TSC complex showed a high potency in cytotoxicity, with an IC_50_ of 0.26 µM for HL60 (human promyelocytic leukemia), 0.62 µM for THP-1 (human monocytic leukemia), 0.09 µM for MDA-MB 231 (human breast adenocarcinoma), and 0.42 µM for MCF-7 (human breast adenocarcinoma) [31].

In another study, Ni chelated with testosterone TSC to form a distorted square planar with ligand as a bidentate NS donor—Ni–bisTSC [44]. Ni–bis(TSC) did not inhibit *E. coli* TopI, but it showed DNA binding affinity similar to ethidium bromide, which results in selective activity against human prostate cancer cells [44].

In summary, the research on metal–TSCs inhibiting Top1B is limited. Some compounds displayed catalytic inhibition, such as [Au(PyCT4BrPh)Cl]Cl, others are interfacial poisons by inhibiting ligation, including Cu(PyCT4BrPh)Cl, Pd–Pyrene–TSC, and Ga(III)–TSC. The cell toxicity results are similar and the IC_50_ is between 0.1 and 10 µM, and some of the metal complexes showed selective activities towards tumor cells.

#### 2.2.2. Inhibition of Type II Top

**Figure 3:**
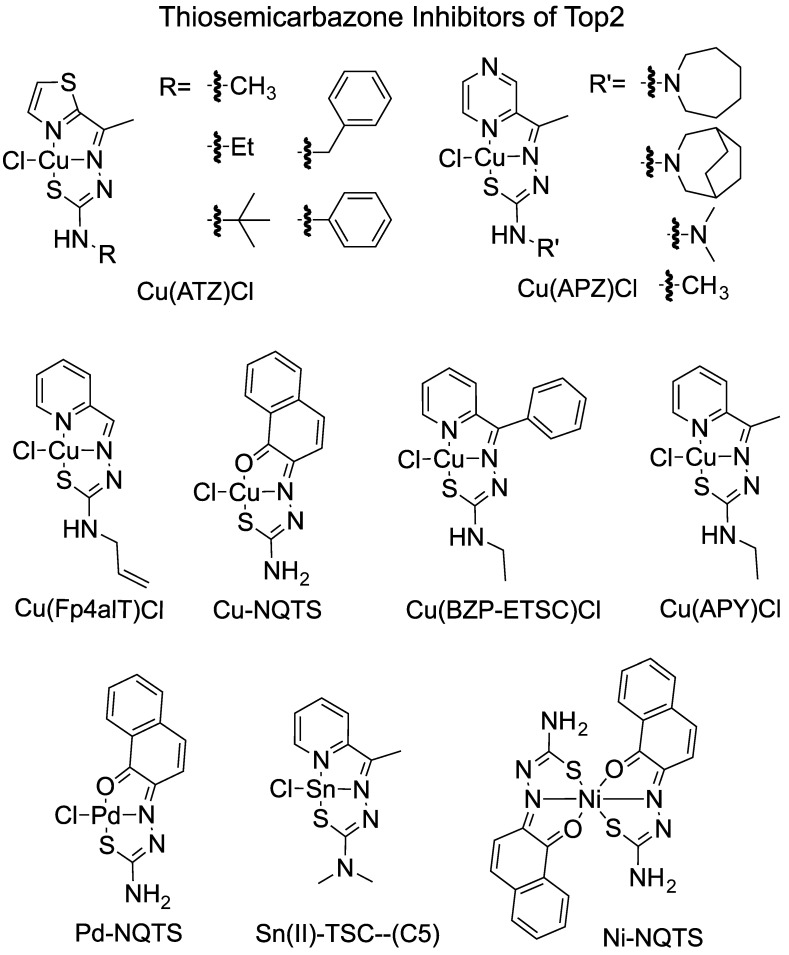
Examples of metal–TSC complexes studied with Top2. Several metal–TSC complexes are shown along with some varying side-chain examples.

Type II Tops are the primary targets for studies of TSC antitumor activity. Multiple metal–TSC complexes showed higher inhibition compared with their ligand counterparts (Figure 3 and Table 3). Cu–TSCs are the most studied and have demonstrated the highest inhibition of Top2.

1. Ni chelated with bis(TSC)

Ni–TSCs were discovered to block a variety of metabolic pathways, including purine synthesis, DNA polymerase, PRPP-amino transferase, IMP dehydrogenase, dihydrofolate reductase, TMP-kinase, and thymidylate synthetase activities, against the L1210 cell line in 1997 [38]. Despite the fact that Ni–TSCs demonstrated several cellular pathways for inhibition, research suggests that Ni–TSCs do not efficiently inhibit Top enzymes [13,38]. Ni(II) coordinates with two TSC ligands, Ni–bis(TSC), which lack the essential square planar structure for Top inhibition [32,38]. The ED_50_ is between 1 and 4 µg/mL against the growth of murine or human leukemias, human HeLa uterine suspended carcinoma, colon adenocarcinoma SW480, KB nasopharynx, lung MB 9812 bronchogenic carcinomas, solid HeLa uterine carcinoma, and rat osteosarcoma [38].

There are some controversies on the inhibition of Ni–TSCs against Top2. Ni-NQTS has very effective antiproliferation activity against the MCF-7 breast cancer cell line with an IC50 of 2.25 μM, better than its copper and palladium counterparts, and it showed inhibition of a Top2α-mediated DNA relaxation assay using a TopoGEN topoisomerase assay kit (Buena Vista, CO) [36]. However, the data are inconsistent with other reported results. For example, a yeast screen did not show that Ni–bisTSC interferes with Tops [13]. Our unpublished results showed that when bisTSCs chelate with metal ions, the metal–bisTSC compounds do not inhibit Top2α (Beckett and Jiang, unpublished). Ni-NQTS were also tested in DNA cleavage assays with Top2α [36]. The results seem to show that Ni-NQTS does not stabilize double-stranded DNA cleavage, but there was a low amount of nicking observed, though it was not quantified [36]. In another study, several Ni–TSC complexes were examined alongside Cu analogs discussed below [47]. Interestingly, Ni(L1)(HL1)Cl, Ni(HL2)_2_Cl_2_, Ni(L3)_2_, Ni(L4)_2_, and Ni(L5)_2_Cl_2_ did not appreciably inhibit Top2α from TopoGEN [47]. Although Ni–TSCs performed profound inhibition against cell proliferation, Top2 may not be the target (or the primary target) for Ni–TSCs [8,9,10].

2. Cu-chelated TSCs

In cell toxicity studies, copper (Cu^2+^)-chelated TSCs are one of the most active groups of metal–TSCs (Table 3) [8,10]. When copper chelates with TSC, it forms a square planar structure with Cu in the middle, which seems to be the crucial structure element for Top2 inhibition [32,33,34], Cu(TSC)s demonstrated greater inhibition compared with their ligands. For example, Cu(TSC)Cl (Compounds **1** and **2**) inhibited Top2 while the corresponding TSC ligands (Compounds **24** and **36**) did not [12]. In general, Cu(TSC) complexes act on Top2 as catalytic inhibitors through inhibiting the ATPase function and inhibiting relaxation.

Another study showed that Cu(TSC)Cl complexes (Compounds **1**–**3**) reduced the DNA cleavage observed in the presence of etoposide, and these compounds alone did not show any stabilization of cleavage complexes [48]. Cu-NQTS inhibited Top2α-mediated DNA relaxation assays and it showed comparable IC_50_ with etoposide in cytotoxicity [36].

Cu(Fp4alT)Cl completely inhibits Top2α without promoting the formation of linear DNA products [37]. Similar results were observed with the other Cu(TSC)Cl complexes in the study [37]. Thus, Cu(Fp4alT)Cl and its family of Cu(TSC)Cl complexes are catalytic inhibitors of Top2α rather than poisons of the enzyme [37].The IC_50_ of Top2α inhibition of Cu(Fp4alT)Cl is 0.3 μM and between 0.6 and 7.2 μM for the rest of the Cu–TSC complexes, while the IC_50_ of Top2α is between 50 and 90 μM for etoposide and 1 and 5 μM for doxorubicin [37]. The cell toxicity for Cu(Fp4alT)Cl is 0.8 μM for the SK-BR-3 cell line and 4.6 μM for MCF-7 cells [37]. The cytotoxicity data for other Cu–TSC complexes are between 0.4 and 12 μM[37]. Cu(L1)Cl, Cu(L2)Cl, Cu(L3)Cl, Cu(L4)Cl, and Cu(L5)Cl_2_ all showed inhibition of Top2α from TopoGEN [47]. Cu(TSC) cation (Complex 1) increased DNA cleavage complexes and inhibited DNA relaxation [49]. It had better efficacy in inhibiting cell growth of the colorectal cancer cells when compared to etoposide [49]. In another study, the complexes [Cu(S,R)-L] and [Cu(R,S)-L] showed inhibition of Top2α relaxation at 300 µM [50]. However, the concentration of inhibition is similar to the ligand TSC and much higher (10–100+-fold) than other Cu(TSC)Cl [50].

Our collaboration worked on a series of Cu(TSC)Cl complexes that demonstrated their inhibition of both human Top2α and Top2β [32,33,34]. The structure–activity relationship of metal–TSCs showed that Cu(II) played a predominant role in the inhibition of Top2 [32,33,34]. The mechanism of Cu(TSC)Cl inhibition on Top2 is complicated. Cu(TSC)Cl inhibited ATP hydrolysis and plasmid DNA relaxation by Top2α and Top2β, which is consistent with these compounds acting as catalytic inhibitors. However, unlike other catalytic inhibitors, Cu(TSC)Cl complexes stabilize the DNA cleavage complexes and increase levels of DNA cleavage, which is the characteristic of interfacial poisons [32,33,34]. In addition, the complexes we tested led to higher levels of double-stranded breaks implying an increase in coordination between the two active sites [32]. The increase in DNA cleavage was not seen in a mutant lacking the ATPase domain [32]. Further, incubation of Cu(TSC)Cl complexes with Top2α or Top2β prior to DNA leads to a progressive inactivation of the enzyme [32,34]. Consistent with this data is the observation that Cu(TSC)Cl stabilizes a closed N-terminal region (ATPase domain) of Top2α or Top2β [34]. The significance of this particular aspect is that the ATPase domains of each half of the homodimer close around DNA in the presence of ATP. Our results demonstrate that the Cu(TSC)Cl complexes that were studied were able to induce closure of this N-terminal gate in a way that stabilized the gate, similar to what is seen with a non-hydrolyzable ATP analog (AMP-PNP) [34].

Although Top2α has been widely used as the molecular target to study Cu(TSC)Cl inhibition, our research found that Cu(TSC)Cl complexes inhibited ATPase and relaxation activity of both Top2α and Top2β [34]. Taken together, the data support the idea that these Cu(TSC)Cl complexes act on or near to the ATPase domain, which is highly similar between both isoforms. Using N-terminally and C-terminally truncated versions of Top2α or Top2β, both resulted in a lack of increased DNA cleavage [32,34]. Interestingly, some Cu(TSC)Cl showed inhibition both of Top1 and Top2, as will be discussed below [45].

Other metal–TSCs also showed inhibition of Top2. Pd-NQTS had an IC_50_ of 13 μM for MCF-7 and inhibited a Top2α-mediated DNA relaxation assay [36]. Cu-NQST is four times more efficient in cytotoxicity compared with Pd-NQST. When chelated with the same ligand, Pd(TSC)Cl seemed to be less active compared with its copper counterpart [33]. Ru(TSC)Cl {[(η-6-p-cymene)Ru(EtATSC)Cl]+ cation}, with a big substrate ring structure, inhibited human Top2α in a relaxation assay [39]. The ruthenium complex of TSC has been tested in a Top2α-mediated DNA relaxation assay and found to inhibit relaxation. The cell toxicity results showed that Ru–TSC complexes showed less or sometimes comparable anti-proliferation activities compared with cisplatin and etoposide [39]. Sn(II)-chelated TSC complexes (C5) inhibited Top2 at 20 µM [40].

**Table 3:** Metal–TSC inhibition of Top2.

Name	Inhibition of Top2	Reference
Nine compounds and their copper complexes	Inhibit human Top2α	[45]
Cobalt (III) chelated with TSC ligand Complex 4	Inhibits human Topo2α-induced DNA relaxation	[41]
Ni–bis(TSC) Complex 1	Does not inhibit isolated Top2 from L1210 cells at 100 µM	[38]
Cu–TSCs (Compound **1** and **2**)	Inhibits isolated Top2 from L1210 cells	[12]
Copper TSC	Inhibits Top2 of L1210 cells with IC_50_ value of 6.25–12.2 µM. Antagonizes the DNA break affect by etoposide.	[48]
Ni-NQTS	Inhibits DNA relaxation	[36]
Cu-NQTS	Inhibits DNA relaxation (TOPOGEN kit)	
Pd-NQTS		
Cu(Fp4alT)Cl and its family of Cu(TSC)Cl	Inhibits relaxation by Top2 at 10 µM Completely inhibits Top2α without promoting the formation of linear DNA products	[37]
Ni–bis(TSC)	No effect in stabilizing DNA breaks	[51]
Cu(TSC)	Stabilizes DNA breaks	[51]
Ni(L1)(H1)Cl, Ni(HL2)_2_Cl_2_, Ni(L3)_2_,Ni(L4)_2_ Ni(L5)_2_Cl_2_	No inhibition of Top2 (TopoGEN)	[47]
Cu(L1)Cl, Cu(L2)Cl, Cu(L3)Cl, Cu(L4)Cl, Cu(L5)Cl_2_	Inhibit Top2 (TopoGEN)	[47]
Complex 1 (CuTSC cation)	Inhibits DNA relaxation and increase DNA cleavage	[49]
Sn(II)–TSC—(C5)	Inhibits Topo2α at 20 µM	[40]
Cu(S,R)L and Cu(R,S)L	Inhibit Top2α relaxation at 300 µM	[50]
[Cu(APY)Cl] and [Cu(APZ)Cl]	Inhibit Top2α from 0.5 µM Increase DNA cleavage Inhibit Top2α ATP hydrolysis No inhibition of ligation by Top2α Pre-incubating compounds with Top2α inactivated the enzyme	[32]

[Cu(APY)Cl] and [Cu(BZP)Cl]	Inhibit Top2β at 5 µM Increase DNA cleavage by Top2β Inhibit Top2β ATP hydrolysis Inhibit ligation by Top2β Pre-incubating compounds with Top2β inactivate the enzyme Stabilized closure of N-terminal Top2α and Top2β clamp	[34]

Cu(BZP)Cl series and	Inhibit Top2α relaxation and increase DNA cleavage	[33]
Cu(ATZ)Cl series		
Pd(BZP)Cl series	Inhibit Top2α relaxation and increase DNA cleavage	[33]

#### 2.2.3. Inhibition of Type I and Type II Top

A few studies have compared the inhibition on Top1B and Top2α. Nine compounds and their copper complexes were investigated against human Top1B and Top2α from TopoGEN [45]. Relaxation assays were quantified to generate an IC_50_. The Cu–TSC complexes were at least 10-fold more effective than the ligand alone [45]. They displayed greater inhibition of Top2α than Top1B [45]. Interestingly, the larger side chain substitutions generally displayed better inhibition of Top2α [45].

Complex 4 of Co(III)–TSC complexes inhibited Top1B-induced and Top2α-induced DNA relaxation, but neither the ligand nor its precursor was able to inhibit either enzyme [41]. Complex 4 did not cause a significant increase in DNA complexes with Top1B or Top2α, which suggests that Complex 4 is a catalytic inhibitor not a poison [41].

## 3. Discussion

**Figure 4:**
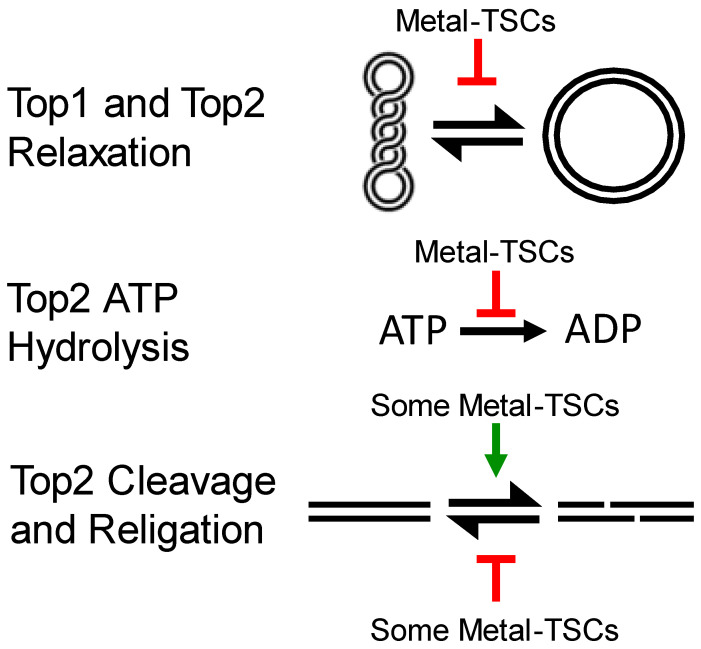
Mechanisms of TSCs against Top1B (Type IB) and Top2α/β (Type IIA). Metal–TSCs appear to inhibit plasmid DNA relaxation and ATP hydrolysis. Some metal TSCs have been shown to increase plasmid DNA cleavage and/or inhibit religation.

TSCs are a broad group of compounds and thus many diverse TSCs have been synthesized and examined. Some of the TSC ligands have been tested in clinical trials such as Triapine and Dp44mT. Although metal–TSCs showed promising results in cytotoxicity and Top inhibition, none of these have been advanced to clinical studies. While TSCs display a broad range of possible mechanisms of action, direct studies on purified Top enzymes have been very helpful in identifying the mechanism of inhibition (Figure 4). Based upon the evidence in the literature, both Type IB (Top1B) and Type IIA (Top2) are affected by various TSCs. This review could not find any evidence of studies with Type IA (Top3) enzymes and TSCs. While Top1B is targeted by some TSCs, far fewer studies have examined Top1 than Top2 enzymes. Far more types of TSCs have been tested against Top2 than Top1B. While the main impact on Top1B appears to be inhibition of relaxation, there are a variety of impacts on Top2 depending on the specific TSC. This is likely due to the more complex reaction mechanism of Top2.

Cu–TSCs have been studied extensively against Top2α. The mechanism showed that Cu–TSCs are catalytic inhibitors that also exhibit some features of interfacial poisons (namely the increase in DNA cleavage levels). Some metal–TSCs inhibited DNA religation by Top1B, but religation of DNA by Top2 is not consistently inhibited by Cu–TSCs. The cleavage complexes created by Top2 are stabilized by the metal–TSC complexes may or may not involve inhibition of religation [32,33,34]. Further, Top2 enzymes generally are more impacted by metal–TSC compounds rather than the ligands alone. There remains a lack of a clear understanding of exactly where and how these compounds are acting. For example, biochemical evidence supports that these compounds can inhibit ATP hydrolysis, but some also increase DNA cleavage. These two mechanisms seem to contradict one another since the general thought is that catalytic inhibitors affect ATP hydrolysis but do not disturb DNA cleavage. Interestingly, some of these compounds appear to stabilize the N-terminal clamp of Top2α and Top2β, which may help clarify the increase in DNA cleavage [34]. For instance, 1,4-benzoquinone is known to increase DNA cleavage and stabilize the N-terminal clamp, likely through a covalent adduction mechanism [34,52,53,54]. Several computational studies indicated that TSC complexes can bind in or around the ATPase domain, which is similar to the mechanism observed with 1,4-benzoquinone. Again, these data are consistent with the observation that N-terminally and C-terminally truncated mutants of Top2α or Top2β are not affected by metal–TSC complexes [32,34]. Together, these data suggest that there may be an increase in coordination between the two active sites of Top2 when metal–TSCs are present, which could result in increased DNA cleavage without true poisoning.

Although there are many molecular modeling studies to predict where TSC binds on Tops, currently no NMR or crystal structure information is available on the exact location of where TSC or metal–TSC binds on Tops. Further structural studies are urgently needed to elucidate the molecular structural information of metal–TSC inhibition on Tops. The mode of interaction of the Cu(TSC)Cl complexes with Tops may be inferred from the literature of TSCs interacting with other molecules. It has been well established that Cu(TSC)+ complexes have been easily synthesized and crystal structures have been produced solved that demonstrate that the complexes can pick up water as a ligand and form five-coordinate complexes [44,45]. Many of the Cu(TSC)Cl complexes are often found in the solid state as dimers, [Cu(TSC)Cl]_2_, which shows a weak bond, dissociable in solution, at the Cu center making it five-coordinate. This structure then forms the square planar four-coordinate complex Cu(TSC)Cl in solution. Also, Cu(TSC)Cl complexes can dissociate the chloride ion (Cl^-^) in aqueous solution to replace Cl^-^ with other ligands and water [46]. These observations indicate that Cu(TSC)Cl complexes can shed Cl- and bond directly with Tops. The observation that they can become five-coordinate indicates that the complex possibly forms two bonds with Tops. This may suggest why structurally similar square planar Pd(TSC)Cl and Pt(TSC)Cl complexes may lose the Cl- ligand to bind to Tops, but they cannot form five-coordinate structures, and thus, are not as potent inhibitors of Tops as the Cu(TSC)Cl complexes.

The ability of metal–TSCs to inhibit Top2α and Top2β suggests that these compounds may have therapeutic potential. However, there are additional considerations that must be addressed before these compounds can be used clinically. First, it is unclear whether inhibition of cell growth can be attributed to Top2 or to other possible mechanisms, known or unknown. Given that the ATPase domain of Top2 is similar to some other enzymes (GHKL ATPase/kinase superfamily), it is possible that metal–TSCs may impact other enzymes as well [55]. Second, the reactivity of these compounds must be explored to determine whether these compounds can covalently interact with proteins and the consequences of this must be considered. Previous data indicate that Top2 can be inactivated through incubation with Cu(TSC)Cl complexes, and the mechanism of this needs to be explored to determine whether this is a specific action against Top2 or could occur more generally leading to unforeseen collateral damage. Also, the specific metal ions that interact with these compounds tend to be d-block metals that can participate in redox reactions. Thus, the extent of that reactivity needs to be considered as the mechanism(s) of action are studied. Third, there is significant structure–activity relationship data available to focus on specific families and classes of TSCs for further development. Fourth, the delivery, bioavailability, and metabolism of these compounds will need to be established and could vary widely depending on the structure of the complex and the metal ion chosen. Much work toward this area has been conducted with the few compounds that have reached clinical trials, and these efforts should help guide the development of additional compounds. In spite of the challenges that lie ahead, these compounds represent a promising area of further research.

## Figures and Tables

**Table 2:** Metal–TSC inhibition of TopI.

Name	Inhibition of TopI	Reference
Cu(PyCT4BrPh)Cl	Inhibits TopI The inhibition is severe with pre-incubation of the compound with TopI Inhibited the cleavage step and partially inhibited religation	[35]
Pd–pyrene–TSC	Inhibits human Top1B at 12.5 µM	[43]
Ga(III)–TSC complex (C4)	Inhibits TopI	[42]
Au(III)(TSC)Cl (complex 1)	Inhibits human Top1B activity starting at 1.5 μM Pre-incubation of Top1B with Complex 1 increased the inhibition	[31]
Ni-bis(TSC)	No inhibition of *E. coli* TopI	[44]
Nine copper complexes	Inhibits TopI	[45]
Cobalt (III)–TSC (Complex 4)	Inhibits TopI-induced DNA relaxation	[41]

## Data Availability

Not applicable.
